# Role of taurine in the central nervous system

**DOI:** 10.1186/1423-0127-17-S1-S1

**Published:** 2010-08-24

**Authors:** Jang-Yen Wu, Howard Prentice

**Affiliations:** 1 Charles E. Schmidt College of Biomedical Science, Florida Atlantic University, Boca Raton, FL 33431, USA

## Abstract

Taurine demonstrates multiple cellular functions including a central role as a neurotransmitter, as a trophic factor in CNS development, in maintaining the structural integrity of the membrane, in regulating calcium transport and homeostasis, as an osmolyte, as a neuromodulator and as a neuroprotectant. The neurotransmitter properties of taurine are illustrated by its ability to elicit neuronal hyperpolarization, the presence of specific taurine synthesizing enzyme and receptors in the CNS and the presence of a taurine transporter system. Taurine exerts its neuroprotective functions against the glutamate induced excitotoxicity by reducing the glutamate-induced increase of intracellular calcium level, by shifting the ratio of Bcl-2 and Bad ratio in favor of cell survival and by reducing the ER stress. The presence of metabotropic taurine receptors which are negatively coupled to phospholipase C (PLC) signaling pathway through inhibitory G proteins is proposed, and the evidence supporting this notion is also presented.

## Introduction

Taurine, 2-amino-ethanesulfonic acid, is one of the most abundant amino acids in mammals [1]. The physiological role of taurine has received considerable attention since the reports that cats fed a taurine deficient diet developed central retinal degeneration [2] and cardiomyopathy [3]. Now, taurine has been shown to be involved in many important physiological functions [for review, see [4]] e.g., as a trophic factor in the development of the CNS [5] and, for instance, kittens from the taurine-depleted mothers exhibit a delay in the migration of cells in the cerebellum and in the visual cortex [5]. It also serves in maintaining the structural integrity of the membrane [6], regulating calcium binding and transport [7,8], as an osmolyte [9,10], a neuromodulator [11], a neurotransmitter [12-18] and a neuroprotector against L-glutamate (L-Glu)-induced neurotoxicity [19,20]. In this article, the role of taurine in the central nervous system (CNS) as a neurotransmitter, a neuro-protective agent and a potent regulator for intracellular calcium homeostasis will be reviewed.

### Taurine as a neurotransmitter

In general, a substance can be accepted as a neurotransmitter if it has fulfilled the following set of criteria: firstly, the substance and/or its synthesizing enzyme has to be present in the suspected neuron, preferably it is concentrated at the nerve terminal; secondly, it is released upon stimulation in a calcium-dependent manner; thirdly, it elicits proper physiological response; fourthly, a specific receptor is present and fifthly, an inactivation mechanism is present to terminate the action of the suspected neurotransmitter. The following lines of evidence have supported the notion that taurine is a neurotransmitter in the mammalian CNS: 1. The presence of a specific enzyme responsible for taurine biosynthesis in the brain, namely, cysteic/cysteine sulfinic acid decarboxylase (CAD/CSAD) which is distinctly different from the GABA-synthesizing enzyme, L-glutamate decarboxylase (GAD) was reported [21,22]. Immunocytochemical studies have revealed the localization of CAD/CSAD in the cell body, dendrite as well as in the nerve terminal [13][17][18][23][24-26]; 2. Release of taurine has been shown to be either calcium dependent or calcium independent [13]; 3. Taurine has been shown to elicit neuronal hyperpolarization presumably through its action by opening the chloride channels in the cerebellum [27] and in the hippocampus [14]; 4. The presence of a specific taurine receptor has been demonstrated. Previously we reported the presence of specific taurine receptors which have Kd in nM range and are distinctly different from GABA_A_, GABA_B_ and glycine receptors since the agonists or antagonists of these receptors have little effect on the binding of taurine to taurine receptors [28]. Similar observations were recently reported by Frosini et al [29]; 5. The presence of a taurine transporter system for inactivation of its function has also been reported [30]. In fact, taurine transporters have been cloned [31] and taurine transporter knock-out transgenic mice have been established [32]. In summary, taurine has fulfilled most if not all of the criteria to be accepted as a neurotransmitter in the mammalian CNS.

### Regulation of intracellular calcium homeostasis

**Figure 1:**
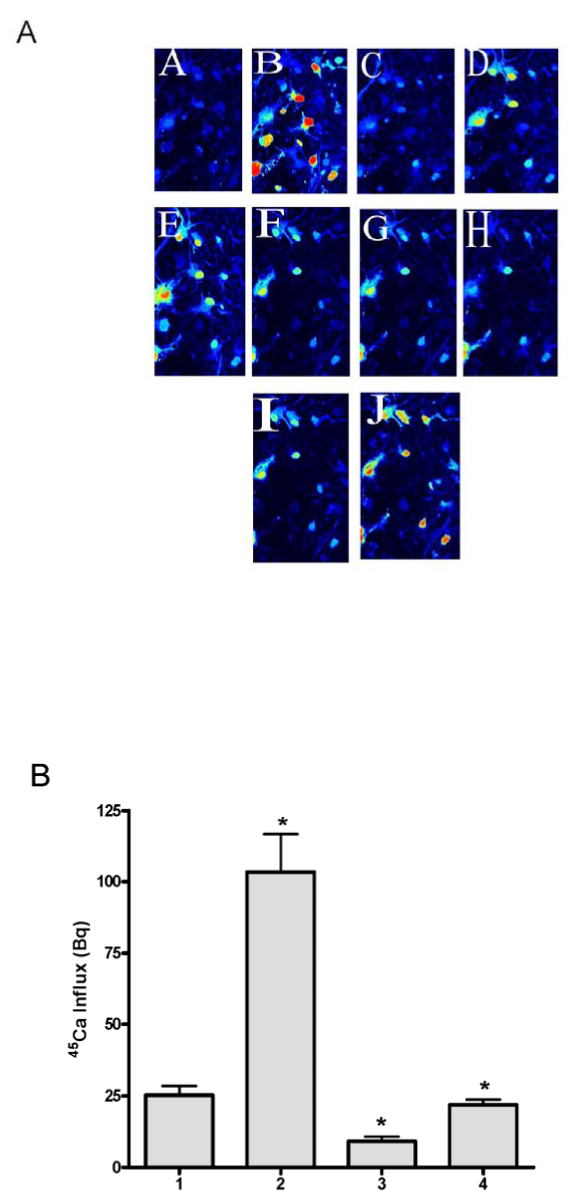
**Effect of taurine on glutamate induced [Ca^2+^]_i_ accumulation (A) confocal study** - A. Baseline; B & J. Glutamate; C, F & I. After washing; D. Nifedipine; E. Nifedipine plus glutamate; G. Taurine; H. Taurine plus glutamate. Color coding indicates [Ca^2+^]_i_, red being the highest and blue the lowest. **(B). Accumulation of ^45^Ca^2+^ influx** - 1. Control; 2. Glutamate; 3. Glutamate plus 25mM taurine; 4. Glutamate plus 5mM taurine.

It is known that the level of intracellular free calcium, [Ca^2+^]_i,_ is maintained at sub-micromolar concentration by calcium sequestering into internal calcium storage pools e.g., mitochondria, endoplasmic reticulum (ER) as well as pumping out to the extracellular space by calcium-ATPase. When neurons are stimulated by glutamate, the [Ca^2+^]_I_ level is elevated due to influx of calcium from extracellular sources through various calcium channels including NMDA receptors, voltage-gated calcium channels (VGCC) such as L-, N- and P/Q-type, reverse mode of Na^+^/Ca^2+^ exchanger as well as release of calcium from the internal calcium storage pools. However, in the presence of taurine, glutamate-induced increase of [Ca^2+^]_i_ is markedly reduced as shown in Fig 1.

**Figure 2:**
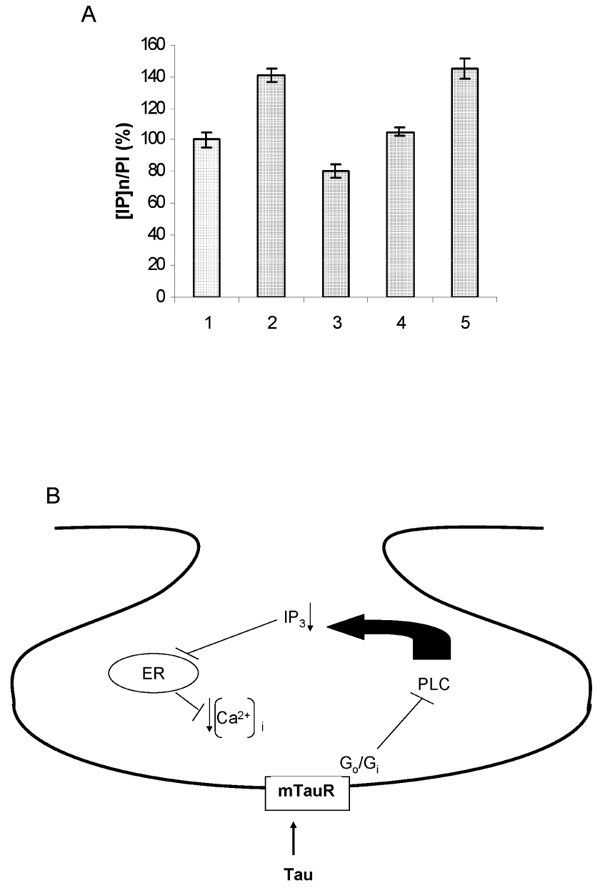
**A. Effect of taurine on PLC activity; B. A proposed model for the mode of action of taurine on the IP3 pathway.A. Effect of taurine on PLC activity.** 1). Control, 2). 0.25mM Glu, 3). 25mM taurine, 4). 25mM taurine plus 0.25mM Glu, 5). 10μM carbachol. The error bar indicates the standard deviation with N=3. Briefly, ^3^H-inositol was used as precursor for [^3^H]-phosphoinositides (PI). Hydrolysis of [PI] to inositol phosphates, [IP]_n_, is an index of PLC activity. The experiments were performed as described previously [45]. Primary neuronal cultures in 35 mm dishes were first labeled with 8μCi of [^3^H, U]-inositol for 24 hours. The cells were washed with fresh medium containing 2mM LiCl (to prevent IP_3_ dephosphorylation) and exposed to either Glu, taurine, or carbachol (a muscarinic cholinergic receptor agonist known to stimulate PLC), as indicated. Phosphoinositides, [PI], and inositol phosphates, [IP]_n_, were determined from the organic phase and aqueous phase, respectively as described [45]. The results are expressed as the ratio of [IP]_n_ to [PI]. The results show that taurine reduces the production of basal level of [IP]_n_ by 20% (column 3) and Glu-stimulated increase of [IP]_n_ production by 40% (column 4). This coupled with the above results suggest that taurine may reduce the basal level or Glu-induced increase of poly-PI turnover through its inhibitory effect on PLC via inhibitory G-proteins e.g. G_i_/G_o_-like proteins. **B. A proposed model for the mode of action of taurine on the IP3 pathway.** Diagram illustrating that taurine’s action on taurine receptors results in an inhibition of PLC activity causing a reduction in IP3 formation thus reducing IP3 mediated release of calcium from internal stores.

We [33] and El Idrissi & Trenkner [20] reported that one of the pathways by which taurine reduced glutamate-induced elevation of [Ca^2+^]_i_ is through inhibition of Ca^2+^ influx via the reverse mode of Na^+^/Ca^2+^ exchanger. At the resting membrane potential, Na^+^/Ca^2+^ exchanger functions to move Ca^2+^ out of the cell. However, under depolarizing conditions such as under glutamate stimulation, it reverses its function to facilitate Ca^2+^ influx [34]. The effect of taurine on Na^+^/Ca^2+^ exchanger has been suggested to be in part due to in part its membrane stabilizing activity [35]. It has been shown that phospholipid N-methylation is associated with a decrease in Na^+^/Ca^2+^ exchanger activity [36]. Hence, taurine-mediated reductions in phospholipid N-methyltransferase activity enhance efflux of Ca^2+^ through the Na^+^/Ca^2+^ exchanger and lower tissue Ca^2+^ content. Taurine also promotes Ca^2+^ efflux via the Na^+^/Ca^2+^ exchanger by increasing [Ca^2+^]_i_ in the vicinity of the exchanger. [35]. In addition to Na^+^/Ca^2+^ exchanger, taurine also inhibits the release of Ca^2+^ from internal pools [37] as well as inhibits various voltage-gated calcium channels (VGCC) such as L-, N- and P/Q-type [38]. Although osmotic stress and taurine treatment were reported to affect various channels such as the Na^+^/Ca^2+^ exchanger, the ATP-sensitive K^+^ channel, the L-type VGCC and the fast Na^+^ channel [39], we believe that in neuronal systems, the effect of taurine on various calcium channels is likely due to a combination of counteraction of glutamate-induced depolarization by taurine as well as receptor-mediated G-protein coupled events. This notion is supported by the following observations: First of all, recently we have shown that when glutamate-induced membrane depolarization is abolished by taurine as measured by voltage-sensitive dye, the VGCC activity is also suppressed [38]. Secondly, Kaczmarek [40] reported that exposure of isolated bag cell neurons to activators of protein kinase C caused an increase in the amplitude of voltage-dependent calcium current. Since taurine can prevent protein phosphorylation, such as of its own synthesizing enzyme, CSAD [41], it is reasonable to believe that taurine also can prevent glutamate-induced VGCC activation by inhibiting phosphorylation of these channel proteins. Thirdly, previously we reported the presence of specific taurine receptors which have Kd in nM range and are distinctly different from GABA_A_, GABA_B_, glycine and glutamate receptors since the agonists or antagonists of these receptors have little effect on the binding of taurine to taurine receptors [42]. Similar observations were also reported by Frosini et al [29]. Furthermore, the binding of taurine to taurine receptors is inhibited by GTP or its non-hydrolyzable GTP analog, [γ-S]-GTP, in a dose-dependent manner [43]. It is believed that binding of GTP to the α-subunit of G-protein promotes the dissociation of G-protein from the receptors resulting in the conversion of receptors back to their low affinity conformation. Fourthly, the inhibitory effect of GTP on taurine receptor binding disappeared once G-proteins were removed from the receptors by treating the membranes with a low concentration of Triton X-100 [43]. Fifthly, Glu-induced elevation of [Ca^2+^]_i_ in the absence of extracellular Ca^2+^ is inhibited by taurine suggesting that taurine inhibits the Glu-induced release of Ca^2+^ from the internal pools [37]. It is of interest that GABA_B_ receptor agonists such as baclofen were reported to inhibit a variety of VGCCs including L-, N- and P/Q-type VGCC through GABA_B_ receptor-coupled inhibitory G-proteins, G_o_ and G_i _[44]. Here we propose that similar to the GABA_B_ receptors, when the metabotropic taurine receptors (mTauR) are activated by taurine, the coupled inhibitory G-proteins e.g. G_o_/G_i_ are then activated resulting in inhibition of VGCCs. Furthermore, we propose that mTauR are negatively coupled to phospholipase C (PLC) through inhibitory G-proteins, e.g. G_o_/G_i_ analogous to the GABA_B_ receptors which are negatively coupled to adenylyl cyclase through inhibitory G-proteins [44]. Activation of taurine receptors by taurine would lead to inhibition of PLC activity (Fig 2), resulting in reduction in IP_3_ formation and hence IP_3_-mediated release of Ca^2+^ from the internal pools.

### Taurine as a neuroprotective agent

**Figure 3:**
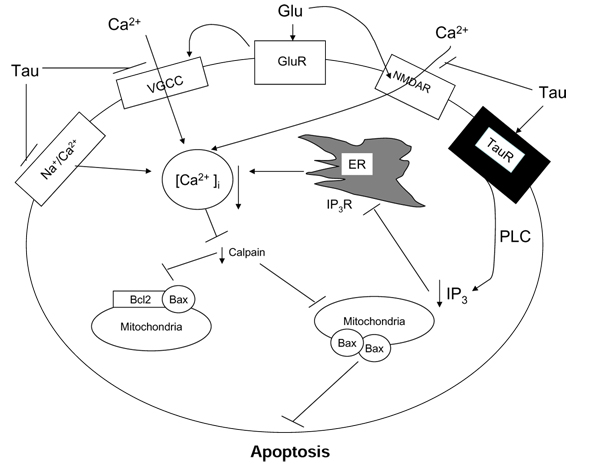
**A model depicting the pathway that taurine exerts its function against glutamate-induced apoptosis.** Taurine’s neuroprotective functions are due to its role in reducing intracellular free calcium concentration and its antioxidative stress capacity. Taurine can shift the ratio of anti-apoptotic Bcl-2 protein and pro-apoptotic Bax protein towards cell survival. As shown in the diagram taurine inhibits glutamate-induced activation of calcium and the subsequent heterodimerization of Bcl-2 and Bax protein resulting in the apoptosis cascade.

One important function of taurine is its neuro-protective function. We [19,33] as well as others [20] have shown that taurine can effectively prevent glutamate-induced neuronal injury in cultured neurons. In addition, we have also demonstrated that taurine can protect against H_2_O_2_-induced cell injury in PC12 cell cultures by reducing H_2_O_2_-induced ER stress (Pan et al 2010, in this issue). It is generally believed that taurine’s neuroprotective functions are due to its role in reducing intracellular free Ca^2+^ concentration, [Ca^2+^]_i_, and its anti-oxidative stress capacity [33,35]. We have recently shown that taurine can shift the ratio of the anti-apoptotic protein, Bcl-2 and the pro-apoptotic protein, Bax, in favor of cell survival [38]. In addition, we have also demonstrated that glutamate-induced activation of calpain is inhibited by taurine resulting in decrease of formation of hetero-dimers of Bcl-2 and Bax and the subsequent release of cytochrome C and the apoptosis cascade [38]. The sequence of events by which that taurine exerts its neuroprotective function can be summarized as follows:

1. Taurine reduces glutamate-induced elevation of [Ca^2+^]_I_ by inhibiting calcium influx from various calcium channels including the reverse mode of Na^+^/Ca^2+^ exchanger, various voltage-gated calcium channels (VGCC) such as L-, N- and P/Q-type, and glutamate NMDA receptors.

2. Taurine inhibits phosphorylation of VGCC resulting in decrease of calcium influx 3. Taurine also reduces the release of calcium from the internal storage pools presumably due to inhibition of phospholipase C.

3. Taurine inhibits glutamate-induced activation of calpain and the subsequent hetero-dimerization of Bcl-2 and Bax protein resulting in inhibition of release of cytochrome C and the apoptosis cascade (Fig 3).

## Competing interests

The authors declare that they have no competing interests.
